# Comparison of Accuracy Between Image-Guided and Freehand External Ventricular Drain Bedside Placement in Association With Time Efficacy

**DOI:** 10.1227/neuprac.0000000000000173

**Published:** 2025-10-13

**Authors:** Iris Charcos, Manisha Koneru, Joseph Ifrach, Kevin Wojcik, Daniel A. Tonetti, Ajith J. Thomas, Corey M. Mossop

**Affiliations:** * Department of Neurosurgery, Cooper University Health Care, Camden, New Jersey, USA;; ‡ Cooper Medical School of Rowan University, Camden, New Jersey, USA

**Keywords:** Surgical navigation systems, Outcomes, Intracranial pressure, External ventricular drain

## Abstract

**BACKGROUND AND OBJECTIVES:**

A common neurosurgical procedure is the placement of an external ventricular drain (EVD), often as a time-sensitive treatment for hydrocephalus and/or increased intracranial pressure. The etiology ranges from traumatic brain injury to spontaneous intracranial hemorrhages, frequently causing change to ventricular anatomy, making them difficult targets. The goal of this study was to determine whether the use of image-guidance for bedside EVD placement improves accuracy and decreases number of passes with the same time-effectiveness.

**METHODS:**

Adult patients with bedside EVD placement for traumatic brain injury or spontaneous intracranial hemorrhages between October 2021 and December 2023 were retrospectively reviewed and dichotomized by electromagnetic image-guided or freehand EVDs. The primary outcome was the rate of suboptimal placement (defined by Kakarla score). The secondary outcomes were rates of multipass EVDs and procedural time.

**RESULTS:**

Of 44 patients included, median age was 59 years (IQR 44-66), and 59.1% were men. Median midline shift was different between image-guided (3.5 mm [IQR 1.5-6.3]) and freehand (0 mm [IQR 0-5]) EVDs (*P* = .0013). With image-guidance, 100% of cases were completed with one pass and optimally placed (Kakarla 1). With freehand placement, 73.5% of cases were completed with one pass, and 88.2% were optimally placed. Median procedural times were similar between image-guided (55 minutes [IQR 50-60]) and freehanded (60 minutes [IQR 48.8-89.3]) placement (*P* = .71). Attributable risk reduction for suboptimal placement with image guidance was 11.76%. For every 9 image-guided EVDs, 1 additional suboptimal EVD placement will be prevented (number needed to treat = 9). Attributable risk reduction for multiple passes with image guidance was 26.5%. For every 4 image-guided EVDs, 1 additional patient will avoid multiple passes for EVD placement (number needed to treat = 4).

**CONCLUSION:**

Image guidance in bedside EVD placement does not require more time in comparison with freehand EVD placement and is associated with fewer passes and higher accuracy.

**ABBREVIATIONS:**
ARR: attributable risk reduction
EVD: external ventricular drain
IVH: intraventricular hemorrhage
NNT: number needed to treat
TBI: traumatic brain injury.

Severe traumatic brain injury (TBI) is a primary cause of death in the United States with an annual incidence of 500 per 100 000 and is associated with increased intracranial pressure (ICP).^1-4^ In patients who present with intracerebral hemorrhage (ICH) with intraventricular hemorrhage (IVH), about 67% develop hydrocephalus.^2^ Placement of an external ventricular drain (EVD) is one of the most commonly performed neurosurgical procedures to treat hydrocephalus and manage elevated ICP in these 2 populations.^5^ In the setting of severe TBI and ICH with IVH, cerebral autoregulation and compliance are frequently affected and small volumes of cerebrospinal fluid (CSF) drainage with EVDs can have a large impact in reducing ICP.^2,3^ Despite this, the reported placement accuracy of EVDs in the literature varies widely from 12% to 83%.^5^ In both populations, accurate placement of the catheter is more difficult because it distorts normal anatomy with smaller ventricles, brain edema, brain shift, and the possibility of external cranial swelling or lacerations.^5-8^

It has been suggested that EVD placement accuracy can be increased by adopting image-guidance techniques as opposed to the traditional freehand placement. A commonly used modality is electromagnetic image guidance which uses computed tomography (CT) or magnetic resonance imaging to create a three-dimensional map of the operative area, allowing a surgeon to track their instrumentation and placement.^9,10^ Image-guided EVD placement uses a similar procedure to freehand, but may require additional setup time.^6^ The aim of this analysis was to determine whether the use of electromagnetic image-guidance for bedside EVD placement improves accuracy and decreases number of passes with similar time effectiveness to traditional freehand EVD placement.

## METHODS

Deidentified data may be made available on reasonable request to the corresponding author. This study was approved by the Cooper Institutional Review Board with waiver of informed consent. This analysis follows the Strengthening the Reporting of Observational Studies in Epidemiology guidelines.

### Population

Adult patients presenting to a high-volume level 1 trauma and comprehensive stroke center between October 2021 and December 2023 were retrospectively reviewed. Patients were identified from ACGME case logs for 2 neurosurgical trainees (I. C. and J. I.) for adult patients requiring twist drill craniostomy for placement of intracranial monitors and subsequently screening for inclusion. Patients met inclusion criteria if EVD placement was performed for severe TBI or spontaneous ICH with IVH; patients were excluded if the procedure was done only for placement of a fiber optic ICP monitor.

### EVD Placement Technique

After the patient is appropriately evaluated for need of CSF diversion, preoperative imaging (CT) is used to identify the appropriate site for catheter placement. Informed consent is obtained for each patient or done with emergent consent. All catheters included in this study were placed by 2 neurosurgical residents at our institution during their second and third years of residency. This addresses and provides balance for the experience level of each resident for this procedure.

**FIGURE 1.**
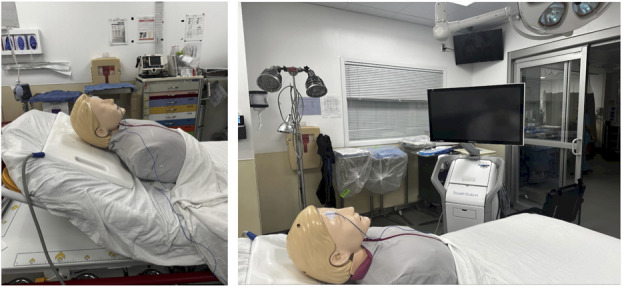
Stealth station set up. Placement of flat emitter plate is shown on the left and positioning for Stealth station is on the right.

**FIGURE 2.**
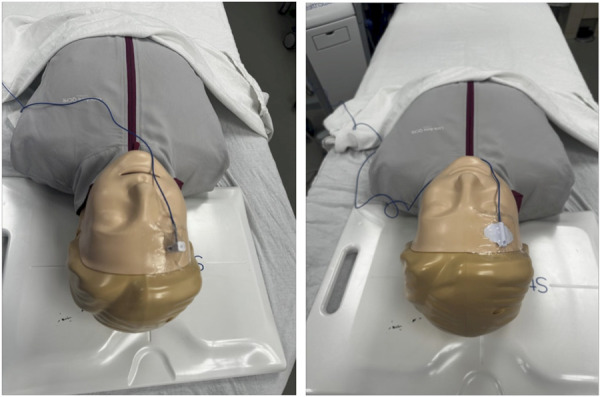
Navigation tracker placement. The images demonstrate placement of navigation trackers using an adhesive dressing. On the left is with an EM skull mounted tracker and on the right with an EM noninvasive tracker.

All image-guided EVDs were placed with the Medtronic AxiEM Electromagnetic StealthStation Surgical Navigation system (Medtronic). If used, the electromagnetic flat emitter is placed underneath the patient's head (Figure 1). Application of the StealthStation EM Noninvasive Patient Tracker using an adhesive dressing (ie, 3M Tegaderm Transparent Film Dressing) for registration allows for more simple reusability (Figure 2). The tracker may then be sanitized for future use. While these trackers are typically meant for one-time use, these application methods allow off-label reuse of the tracker for cost-effectiveness. The patient is registered to the corresponding imaging on the StealthStation and a plan created for EVD insertion site and target (ipsilateral Foramen of Monroe). For imaging, we used thin-slice CT obtained with initial standard imaging at our institution instead of navigational sequencing.

Site of insertion is identified by measuring Kocher's point. If image guidance is being used, the insertion point is confirmed with navigation at one centimeter in front of the coronal suture in the midpupillary line. When using image guidance, the stylet is used at each step to confirm accurate insertion site. The ventricular catheter was then placed perpendicular to the skull. With image guidance, the catheter is shortened to fit over the stylet and any metal retractors removed to prevent interference. The catheter with inserted stylet is then guided in following the predetermined trajectory. In every case, the catheter's position is confirmed with spontaneous flow of CSF and subsequently by postprocedural CT.

### Data Collection

Demographic and clinical data were collected along with radiological records including noncontrast head CT for all patients, which was retrospectively reviewed to ascertain shifting of the ventricular system. Procedural characteristics were assessed and tallied including whether the procedure involved electromagnetic image-guided or freehand EVD placement, the number of passes, placement location on postprocedural CT, and procedural time. Procedural time was defined to incorporate the entire time used for placement of the EVD. This includes the time elapsed from setup of the apparatus, registration of the image-guidance system in image-guided EVD placement procedures until the conclusion of the procedure. Postprocedural noncontrast head CT was reviewed to assess success of EVD placement per the Kakarla grading system. This grading system classifies catheter positioning into 3 grades based on postoperative imaging. Grade 1 indicates optimal placement in the ipsilateral frontal horn or third ventricle for effective drainage. Grade 2 represents functional but nonideal placement in the contralateral ventricle or noneloquent cortex. Grade 3 denotes suboptimal placement in eloquent cortex or nontarget spaces, potentially leading to complications.

### Outcomes

The primary outcome was the rate of suboptimal ventriculostomy placement (defined as Kakarla grades 2-3). The secondary outcomes include the rate of requiring multiple passes for EVD placement and procedural time.

### Statistical Analysis

Continuous variables were summarized as medians with IQRs; categorical data were summarized as frequencies. The population was dichotomized into image guided and freehand EVD placement groups for comparisons. Differences between groups were analyzed using Student *t*-test, Wilcoxon rank sum test, and χ^2^ test. The attributable risk reduction (ARR) and number needed to treat (NNT) using image guidance compared with freehand techniques for the outcomes of suboptimal placement and multipass EVD placement were calculated. Statistical significance was specified at 0.05. Missing data were minimal and not imputed. Analyses were conducted in JMP Pro v. 17.0.0 (SAS Institute Inc).

## RESULTS

### Demographics

Of 44 patients included, median age was 59 years (IQR 44-66), and 59.1% were men (Table). Freehand EVDs were placed in 77.3% (34/44) of patients, and image guidance was used in 22.7% (10/44) of patients (Table). Median midline shift was significantly greater in the image-guided (3.5 mm [IQR 1.5-6.3]) than freehand (0 mm [IQR 0-0.5]) EVD groups (*P* = .001, Table).

**TABLE.** Comparison of Patients With Image Guided and Freehand External Ventricular Drains

Variable	All (n = 44)	Image guided (n = 10)	Freehand (n = 34)
Age (y), median (IQR)	59 (44-66)	44 (30-50)	60 (50-67)
Male, n. (%)	26 (59.1%)	7 (70.0%)	19 (55.9%)
Placement location, n. (%)			
Right frontal	29 (65.9%)	6 (60.0%)	23 (67.6%)
Left frontal	14 (31.8%)	3 (30.0%)	11 (32.4%)
Left parieto-occipital	1 (2.3%)	1 (10.0%)	0 (0%)
Midline shift (mm), median (IQR)	0 (0-3)	3.5 (1.5-6.3)	0 (0-0.5)
Total GCS, median (IQR)	7 (6-10)	7 (3-8)	8 (6-11)
Eye opening score	2 (1-3)	1 (1-2)	2 (1-3)
Verbal response	1 (1-1)	1 (1-1)	1 (1-3)
Motor response	5 (4-5)	5 (1-5)	5 (4-6)
Procedure time (min), median (IQR)	60 (51-74)	56 (50-60)	60 (49-89)
Number of passes, median (IQR)	1 (1-1)	1 (1-1)	1 (1-2)
Single pass placement, n. (%)	35 (79.5%)	10 (100%)	25 (73.5%)
Karkala grade, n. (%)			
1 (optimal)	40 (90.9%)	10 (100%)	30 (88.2%)
2 or 3 (suboptimal)	4 (9.1%)	0 (0%)	4 (11.8%)

GCS, Glasgow Coma Scale.

### Procedural Outcomes

Median procedural times were not significantly different between image-guided (56 minutes [IQR 50-60]) and freehanded (60 minutes [IQR 49-89]) placement (*P* = .71, Table). All cases were completed with one pass in image-guided placement, while 73.5% (25/34) of cases were completed with one pass with freehand placement (Table). All EVDs placed with image guidance were optimally placed (Karkala Grade 1), while 88.2% of freehand EVDs were optimally placed (Table).

The ARR for suboptimal EVD placement with image-guided placement was 11.76%. For every 9 image-guided EVDs, 1 additional suboptimal EVD placement will be prevented (NNT = 9). The ARR for needing multiple passes with image-guided placement was 26.5%. For every 4 image-guided EVDs, 1 additional patient will avoid multiple passes for EVD placement (NNT = 4).

## DISCUSSION

In the literature, freehand EVD placement has been shown to be less accurate than image-guided placement.^5-8,11^ Most studies focus on comparing freehand and image-guided EVD placement in non-TBI or mixed cohorts. Mixed cohorts are more common in the literature and report similar proportions of freehand misplacement of about 20%-30%.^6^ AlAzri A et al conducted a retrospective comparison of freehand and image-guided (CT) EVD placement in severe TBI and found the image-guided placement accuracy was 94.7% (Kakarla Grade 1) while the placement accuracy of the freehand group was 57.1%.^6^ This is compared with Kakarla et al^12^ reporting a misplacement rate of 43.7% in their TBI cohort. Other studies have also commented on procedural time between freehand and image-guided EVDs with varying conclusions from no difference to an increase in times.^5-7^

### Key Results

Our study demonstrates that the use of electromagnetic image guidance for EVD placement in patients with severe TBI and ICH with IVH significantly improves placement accuracy while maintaining procedural efficiency. With 100% optimal placements and single-pass success rates in the image-guided group, our findings align with previous literature that suggests image guidance enhances the precision of EVD placements in complex neurosurgical contexts.^5-8,11,13.^

**FIGURE 3.**
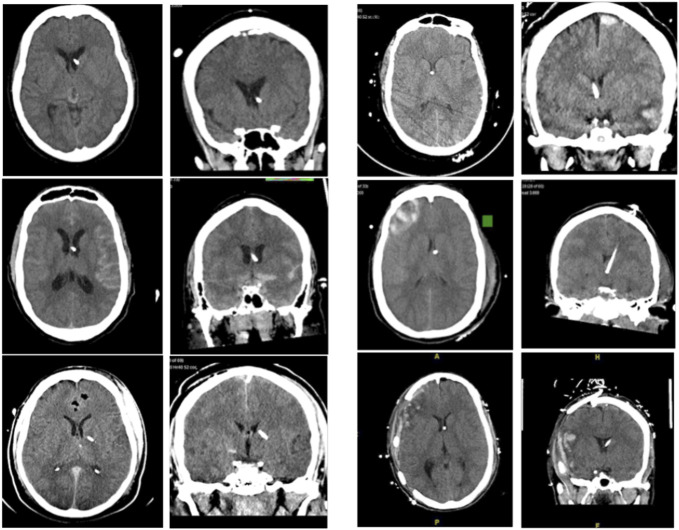
Cases demonstrating freehand EVD placement vs image-guided EVD placement. Freehand EVDs are shown on the left and image-guided EVDs are on the right. EVD, external ventricular drain.

The marked difference in suboptimal placement rates between image-guided (0%) and freehand techniques (11.8%) underscores the challenges associated with freehand placement, particularly in the aforementioned populations, where factors such as brain edema and anatomical distortions complicate traditional methods.^14,15^ This was seen in our patient population as well (Figure 3). Notably in our study, there was a statistically significant difference in midline shift between the 2 groups, with it being greater in patients in which there was use of image guidance for EVD insertion. This would suggest that despite patients having a more distorted anatomy, accuracy, and time efficacy were not negatively affected when using image guidance. The reported placement accuracy of image-guided techniques reinforces the notion that integrating advanced imaging technologies can mitigate the risks associated with EVD misplacement, which can lead to significant morbidity and additional surgical interventions.

In our approach, we do not use formal stealth imaging in our procedure, yet we are able to achieve satisfactory placement and registration in scans with approximately 350 slices that notably excludes the tip of the nose. Use of suboptimal navigation imaging is mitigated by relocating the first touchpoint during registration to distal most portion of the nose available on the scan along with careful tracing registration. This underscores the effectiveness of our approach in maintaining accuracy without relying on formal stealth imaging techniques. This also minimizes any lag time that may occur from repeating imaging with STEALTH sequencing before placing the EVD. In addition, aside from the stylet, which is a single-use item, the remaining equipment required for stereotactic registration is reused. The nonsterile tracer pointer is easily sterilized with antimicrobial wipes, and the noninvasive patient tracker is cleaned in a similar fashion. The tracker is applied between 2 small tegaderm dressings to prevent soiling. This practice contributes to cost efficiency and reduces procedural waste without compromising the reliability of the hardware. It is also worth noting that the stylet, at a cost of $568, represents the only additional expense incurred per procedure. This cost could be an avenue for future study, particularly in evaluating the overall cost effectiveness of this method.

Interestingly, both techniques exhibited comparable procedural times—56 minutes for image-guided and 60 minutes for freehand placement—indicating that the perceived complexity of image guidance does not detract from the efficiency of the procedure. We attribute this rapid procedural time to several aspects of our workflow. A designated travelling navigation station is available and readily usable in all the trauma and critical care settings of our institution. In addition, use of rapid wireless imaging transfer through our PACS along with expedited decision making regarding the use of image-guided EVD placement allow for reduction of lag time through preloading of imaging to the Stealth Station even while the patient completes their full battery of admission CT imaging. Procedural time is measured including setup and registration time. The median procedural time of both techniques suggests that adopting image guidance could be seamlessly integrated into clinical practice without adding substantial delays, a crucial consideration in emergency settings. Even with the current ongoing development in artificial intelligence and augmented reality technologies, stereotactic navigation remains widely available in most institutions, including smaller neurosurgical facilities making it a potentially more adoptable practice. To enhance the efficiency of EVD placement, it is essential to maintain ready accessibility of the Stealth navigation system and ensure that residents and intensive care unit teams are well-informed about the location and proper use of AxiEM equipment. Establishing a streamlined workflow through equipment organization, staff training, and proactive imaging integration will further optimize efficiency and patient care.

Our findings further reveal a notable reduction in the need for multiple passes in the image-guided cohort (0% vs 26.5% for freehand). This not only reflects the technical advantages of image guidance but also suggests potential benefits in reducing patient exposure to the risks associated with multiple catheter insertions, such as infection and additional trauma.

### Limitations

Despite these promising results, our study has limitations, including its retrospective design and relatively small sample size. The study is limited also in that it is a single site, with 2 junior resident operators. Future research should focus on larger, prospective multicenter trials to validate these findings across diverse clinical settings. In addition, exploring the integration of advanced technologies, such as augmented reality and artificial intelligence, could further enhance the efficacy and safety of EVD placements in challenging clinical scenarios.

## CONCLUSION

In this retrospective analysis of neurosurgical residents placing ventriculostomies in severe TBI and ICH, electromagnetic image-guided EVD placement was more accurate and required similar procedural time as freehand EVD placement.
